# Third-Line Palliative Systemic Therapy for Advanced Biliary Tract Cancer: Multicentre Review of Patterns of Care and Outcomes

**DOI:** 10.3390/cancers15113047

**Published:** 2023-06-03

**Authors:** Simon Gray, Octave Letissier, Constance d’Abrigeon, Dinakshi Shah, Stephen Wardell, Olusola Faluyi, Angela Lamarca, Richard A. Hubner, Julien Edeline, Juan W. Valle, Mairéad G. McNamara

**Affiliations:** 1 Department of Medical Oncology, The Christie NHS Foundation Trust, Wilmslow Rd., Manchester M20 4BX, UK; 2 Department of Molecular and Clinical Cancer Medicine, Faculty of Health and Life Sciences, University of Liverpool, Ashton St., Liverpool L69 3GB, UK; 3 Department of Medical Oncology, The Clatterbridge Cancer Centre NHS Foundation Trust, Pembroke Pl, Liverpool L7 8YA, UK; 4 Centre Eugène Marquis, Av. De la Bataille Flandres Dunkerque-CS 44229, CEDEX, 35042 Rennes, France; 5 Department of Oncology, Oncohealth Institute, Jimémez Díaz University Hospital, Av. de los Reyes Catolicos 2, 28040 Madrid, Spain; 6 Division of Cancer Sciences, University of Manchester, Oxford Rd., Manchester M13 9PL, UK

**Keywords:** biliary tract, cholangiocarcinoma, gallbladder, ampulla of Vater, third-line, systemic therapy, chemotherapy, targeted therapy

## Abstract

**Simple Summary:**

Biliary tract cancer (BTC) carries a poor prognosis. Most patients with BTC will require ‘systemic’ therapy, treating cancer throughout the body to control the disease and extend life while maintaining or improving the quality of life, but not to cure. There is no standard third-line systemic therapy, and few patients remain fit to receive three lines of treatment. This study assessed 97 patients from three academic centres who received three lines of palliative systemic therapy for BTC. Median survival after starting third-line treatment was 6.4 months, and from first-line treatment it was 26.9 months. The region of the biliary tract in which the cancer originated did not significantly affect prognosis. The 10 patients with an identified molecular change ‘driving’ the cancer, who received third-line treatment targeting that change, survived longer (12.5 months, versus 5.9 months for all other included patients). This study provides a benchmark for future clinical trials within BTC.

**Abstract:**

Phase 3 trials have established standard first-line (1L) and 2L systemic therapy options for patients with advanced biliary cancer (ABC). However, a standard 3L treatment remains undefined. Clinical practice and outcomes for 3L systemic therapy in patients with ABC were therefore evaluated from three academic centres. Included patients were identified using institutional registries; demographics, staging, treatment history, and clinical outcomes were collected. Kaplan–Meier methods were used to assess progression-free survival (PFS) and overall survival (OS). Ninety-seven patients, treated between 2006 and 2022, were included; 61.9% had intrahepatic cholangiocarcinoma. At the time of analysis, there had been 91 deaths. Median PFS from initiating 3L palliative systemic therapy (mPFS3) was 3.1 months (95%CI 2.0–4.1), while mOS3 was 6.4 months (95%CI 5.5–7.3); mOS1 was 26.9 months (95%CI 23.6–30.2). Among patients with a therapy-targeted molecular aberration (10.3%; n = 10; all received in 3L), mOS3 was significantly improved versus all other included patients (12.5 vs. 5.9 months; *p* = 0.02). No differences in OS1 were demonstrated between anatomical subtypes. Fourth-line systemic therapy was received by 19.6% of patients (n = 19). This international multicentre analysis documents systemic therapy use in this select patient group, and provides a benchmark of outcomes for future trial design.

## 1. Introduction

Biliary tract cancer (BTC) is a heterogeneous group of malignancies comprising cholangiocarcinoma arising from the bile ducts (divided into intrahepatic, perihilar, or distal—iCCA, pCCA, or dCCA, respectively), gallbladder carcinoma (GBC), and ampulla of Vater carcinoma (AVC); pCCA and dCCA have historically been collectively referred to as extrahepatic (e)CCA, but are now considered clinicopathologically distinct entities [1]. Analysis of data from the United States suggests that incidence rates are comparable between iCCA, eCCA, and GBC, while AVC is less common (1.18, 1.02, 1.13, and 0.74 per 100,000, respectively) [2,3,4]. The majority of patients with BTC progress to a locally advanced, recurrent, or metastatic state during their disease course (advanced biliary cancer, ABC), and therefore require considerations of palliative systemic therapy [5].

The landmark United Kingdom (UK)-based ABC-02 trial established cisplatin/gemcitabine (Cis/Gem) as standard-of-care first-line (1L) palliative systemic therapy for BTC, affording a median overall survival (mOS) of 11.7 months [6]. While ABC-02 included patients with Eastern Co-operative Oncology Group Performance Status (ECOG PS) of 0–2, a more recent trial included only patients with ECOG PS 0–1 utilised Cis/Gem plus placebo as a comparator arm, with a resulting mOS of 13.0 months [7]. More recently, Cis/Gem with durvalumab has been shown to improve OS in patients with ABC in a 1L setting [8]. The ABC-06 and Korean NIFTY trials have established 5-fluorouracil (5-FU)/oxaliplatin (FOLFOX) and 5-FU/liposomal irinotecan as 2L treatment options [9,10].

Targeted therapies have also become increasingly utilised for distinct subsets of patients with ABC. Targeted therapies with Food and Drug Administration (FDA) approval within BTC include pemigatinib and infigratinib for patients with BTC with fibroblast growth factor receptor 2 (*FGFR2*) fusions [11,12], and ivosidenib for patients with isocitrate dehydrogenase 1 (*IDH1*) mutations [13]—both molecular aberrations are found almost exclusively in patients with the iCCA subtype [14,15]. Further promising trial data are available for patients with BTC harbouring neurotrophic tropomyosin receptor kinase fusions treated with larotrectinib [16], those with human epidermal growth factor-2 overexpression or amplification treated with trastuzumab and pertuzumab [17], and patients with *BRAF V600E* mutations treated with dabrafenib and trametinib [18]. Pembrolizumab also has site-agnostic FDA approval for the treatment of microsatellite instability-high [19] or tumour mutation burden-high disease [20], which are found in BTC, although only rarely [21,22].

Large Phase 3 trials have found benefit for the use of S-1 (Tegafur/gimeracil/oteracil) in 1L treatment of ABC, in terms of both non-inferiority to Cis/Gem when cisplatin is replaced with S-1, and superiority to Cis/Gem alone when S-1 is added [23,24]. However, S-1 has not been evaluated in Western populations and its use is not currently considered standard-of-care in these patients.

There is no standard 3L systemic treatment for patients with ABC; thus, this analysis aimed to identify practice in systemic therapy selection among the select group of patients who received 3L palliative treatment in these institutions.

## 2. Materials and Methods

### 2.1. Study Overview

This retrospective, multicentre analysis was performed across three sites: The Christie NHS Foundation Trust (CFT) in Manchester, UK; the Clatterbridge Cancer Centre (CCC) in Liverpool, UK; and Centre Eugène Marquis (CEM) in Rennes, France. This project received Quality Improvement & Clinical Audit Committee approval (Reference: SE18/2842).

### 2.2. Patient Selection

Patients were identified by reviews of the institutional registries at each of the three study sites. Patients were included if they were: over 18 years of age; had a diagnosis of intrahepatic, perihilar, or distal cholangiocarcinoma, gallbladder carcinoma, or ampulla of Vater carcinoma (patients with duodenal adenocarcinoma were excluded); ABC, assessed as incurable by treatment providers; oncological care (consultation and/or treatment) received at one of the three study sites with all prior treatments and overall survival known to the relevant study site; receipt of three lines of systemic therapy in the palliative setting. Chemoradiotherapy, locoregional therapies, and adjuvant therapy—including in the setting of early post-operative recurrence—were not included as lines of treatment. Re-challenge treatment on progression was permitted, provided that imaging at the end of initial treatment did not demonstrate disease progression; re-challenge could be as either 1L or 2L systemic therapy.

### 2.3. Statistical Analysis

The primary endpoint of this analysis was OS from the date that the first dose of 3L systemic therapy was received (OS3). Secondary endpoints included: systemic therapies used in each line, OS from initiation of 1L and 2L palliative systemic therapies (OS1; OS2), and progression-free survival from the initiation of each line of palliative systemic therapy (PFS1/2/3). In determining PFS, progression was recorded as defined by the treating clinical team (clinically and/or radiologically) and was not strictly required to be progression as per Response Evaluation Criteria in Solid Tumours 1.1 [25]. The imaging interval for patients receiving standard of care treatment was approximately 12-weekly, and may have been more frequent for those enrolled in clinical trials. Exploratory objectives included assessing the relationship between OS, PFS3, and choices of systemic therapy according to anatomical subgroup. Patients with ongoing treatment were censored at the data cut-off time of 1 August 2022; patients with unknown vital status or who survived off-treatment were censored at the last known follow-up.

All statistical analyses were performed using IBM SPSS Statistics v28.0.1.1. Kaplan–Meier methods, with 95% confidence intervals (CI) at specific time-points, were used to describe the OS and PFS endpoints; the log-rank test was used for comparisons between anatomical subtypes. Descriptive statistics (range, mean, median, standard deviation, and 95% CI) were used as appropriate to describe pathological and clinical features. All *p*-values were two-sided, with *p*-values of <0.05 taken to indicate significance.

## 3. Results

### 3.1. Patient Characteristics

Ninety-seven patients who received at least one dose of 3L systemic therapy for advanced BTC between 2006 and 2021 were included. Table 1 displays patient demographics and clinicopathological disease characteristics. At CFT, 4.7% of patients who started 1L chemotherapy for ABC subsequently received three lines of palliative systemic therapy (n = 40 received 3L palliative systemic therapy); for CEM, this proportion was 15.7% (n = 48), and for CCC, this proportion was 2.0% (n = 9).

### 3.2. Systemic Therapy Regimens

In the first palliative systemic treatment line, the majority of patients (89.7%) received a combination of gemcitabine (Gem) with platinum (cisplatin or carboplatin); a small proportion of patients received targeted therapy or immunotherapy within the context of a clinical trial, alongside Cis/Gem chemotherapy (and were counted within the ‘Other’ category in Table 1). Second-line therapy was mainly composed of FOLFOX or 5-FU/irinotecan chemotherapy, but therapy remained Gem-based in a significant proportion of patients. In 2L, two patients received immuno- or targeted therapy (durvalumab plus tremelimumab, and avelumab plus regorafenib), with the remainder receiving cytotoxic chemotherapy alone.

The third-line systemic therapies utilised are displayed in Table 2. A broader range of therapies was received than for 1L or 2L therapies, including a significant minority of targeted therapies, which were guided by the results of molecular profiling studies—specifically, the 10.3% of patients who received either an FGFR inhibitor (futibatinib n = 3, pemigatinib n = 3, and roblitinib n = 1), ivosidenib, or the MET inhibitor APL 101. Nineteen patients (19.6%) went on to receive at least one dose of fourth-line systemic therapy. Re-challenge of Gem/platinum treatment in either 2L or 3L was received by thirty-five patients (36.1%).

### 3.3. Overall Survival

**Figure 1:**
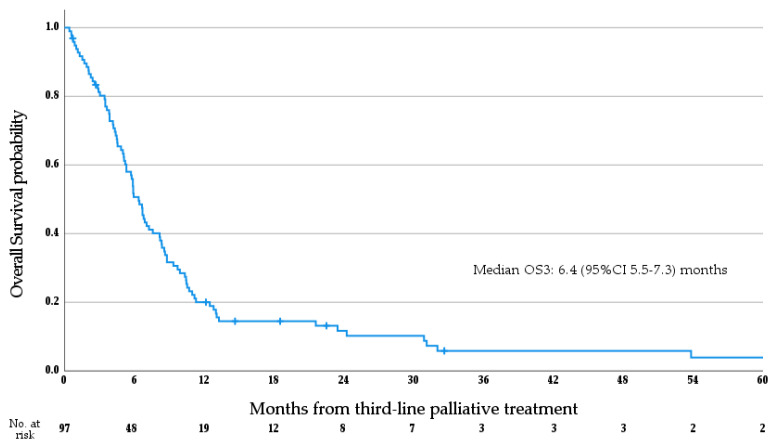
Overall survival from the initiation of third-line palliative systemic therapy.

**Figure 2:**
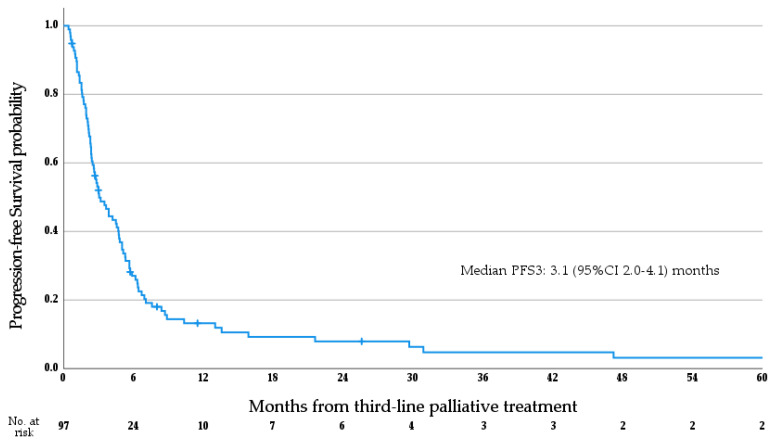
Progression-free survival from the initiation of third-line palliative systemic therapy.

Median follow-up from receipt of the first dose of 3L systemic therapy was 26.2 months (range 4.4–179.0 months). At the time of data cut-off, there had been a total of 91 deaths. Median OS3 for the whole cohort was 6.4 months (95%CI 5.5–7.3 months; see Figure 1). Subgroup analysis via log rank test determined that patients treated with molecularly targeted agents had significantly prolonged OS3 versus patients who were not (median OS 12.5 vs. 5.9 months; *p* = 0.02), while an age greater than 60 at diagnosis (*p* = 0.489) and the presence of metastasis at diagnosis (*p* = 0.461) were not significantly associated with OS3. Median PFS3 for the whole cohort was 3.1 months (95%CI 2.0–4.1 months; see Figure 2).

**Figure 3:**
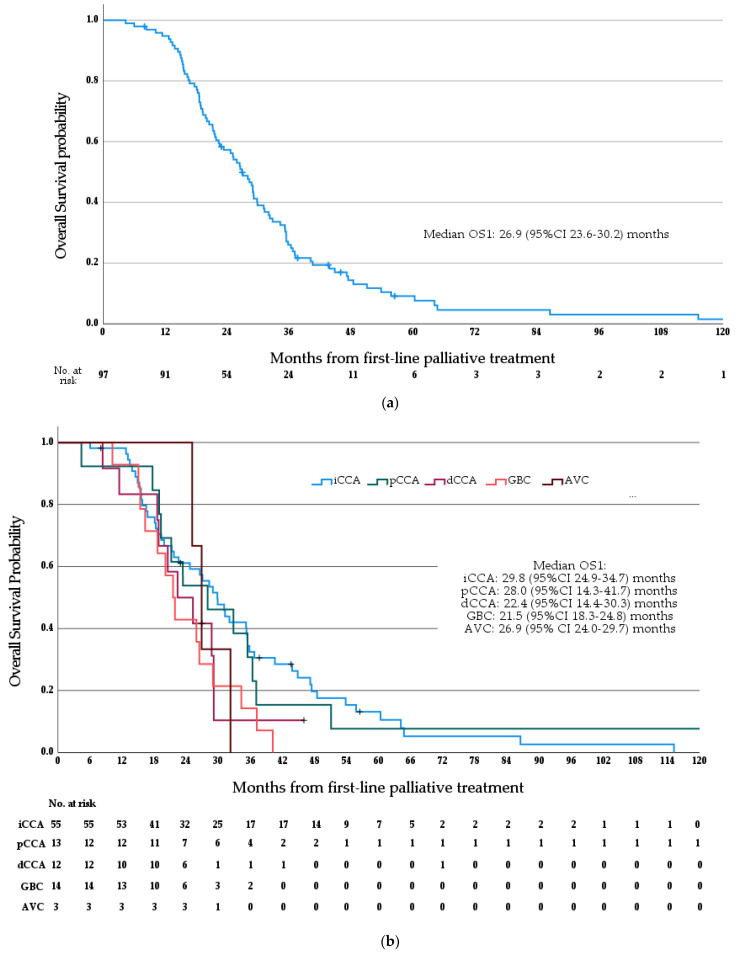
(**a**) Median overall survival from the initiation of first-line palliative systemic therapy. (**b**) Median overall survival from the initiation of first-line palliative systemic therapy, stratified by anatomical subtype.

**Figure 4:**
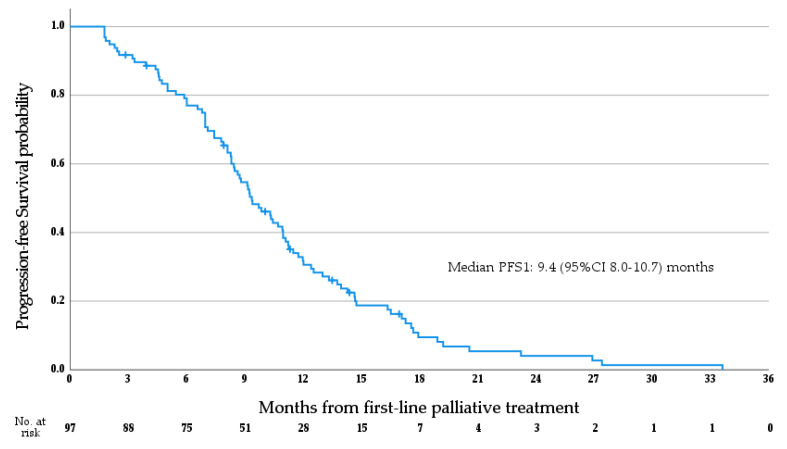
Progression-free survival from the initiation of first-line palliative systemic therapy.

**Figure 5:**
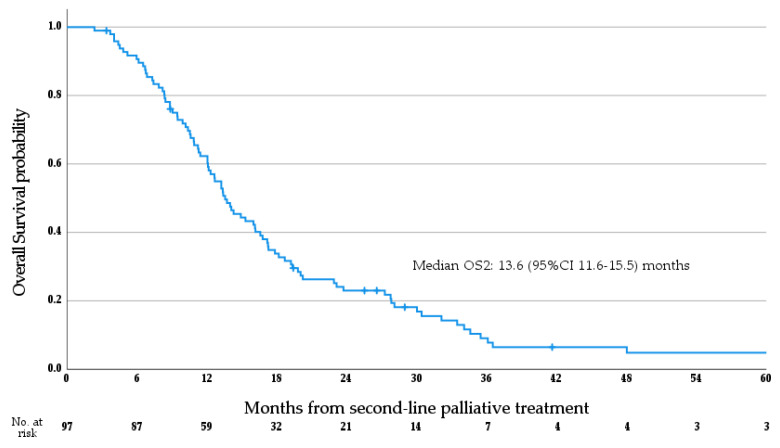
Overall survival from the initiation of second-line palliative systemic therapy.

**Figure 6:**
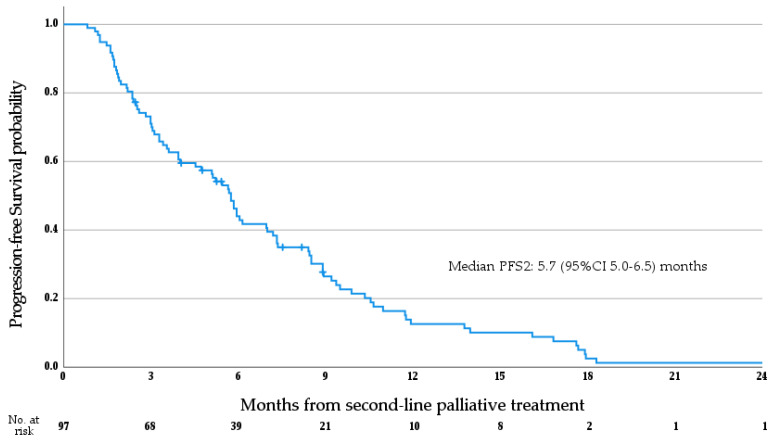
Progression-free survival from the initiation of second-line palliative systemic therapy.

Median OS1 for the whole cohort was 26.9 months (95%CI 23.6–30.2 months; see Figure 3a). Median OS1 stratified by anatomical subtype is as follows (see Figure 3b)—iCCA 29.8 months (95%CI 24.9–34.7 months), pCCA 28.0 months (95%CI 14.3–41.7 months), dCCA 22.4 months (95%CI 14.4–30.3 months), GBC 21.5 months (95%CI 18.3–24.8 months), and AVC 26.9 months (95% CI 24.0–29.7 months); no significant difference in OS1 was observed between anatomical subtypes (*p* = 0.171, log rank test), and no differences were seen between patients with iCCA and non-iCCA ABC (*p* = 0.103, log rank test). Median PFS1 for the whole cohort was 9.4 months (95% CI 8.0–10.7 months; see Figure 4). Median OS2 for the whole cohort was 13.6 months (95% CI 11.6–15.5 months; see Figure 5). Median PFS2 for the whole cohort was 5.7 months (95% CI 5.0–6.5 months; see Figure 6).

Patients who had undergone a prior curative-intent resection had longer mOS from the time of their resection (42.8 months; 95% CI 37.0–48.6 months) compared with mOS from time of first palliative systemic therapy in those who did not undergo a curative-intent resection (22.6 months; 95% CI 16.5–28.6 months; *p* < 0.001, log rank test). Notably, no patients underwent curative-intent resection after the initiation of 1L palliative systemic therapy.

Supplementary Table S1 stratifies patients by year of receipt of 1L systemic therapy; a positive correlation between the year and number of included patients was observed (r = 0.40). This correlation is strengthened to r = 0.6906 when omitting the single included patient who received 1L palliative systemic therapy in 2021, on the grounds that the number of patients who subsequently receive 3L therapy from 2021 may increase substantially in view of shorter follow-up at data cut-off.

Supplementary Table S2 documents fourth-line systemic therapy use among patients who had documented progression through 3L systemic therapy prior to their death—66 patients (68.0%) had documented progression prior to their death, and 19 of these received at least one dose of fourth-line systemic therapy. Of these, 94.7% received cytotoxic chemotherapy, and only 3 received single-agent chemotherapy (Gem), with all others receiving combination treatment; 1 patient received a combination of targeted therapy and immunotherapy.

## 4. Discussion

This multicentre, retrospective analysis of 3L systemic therapy in patients with ABC represents a highly select group of patients, especially given previous retrospective observations that only around one-quarter of patients with ABC receive 2L systemic therapy [24]. The authors are unaware of any published studies assessing the proportion of all patients receiving palliative systemic therapy in a real-world clinical practice setting who receive 3L treatment. The majority of patients in the present analysis received cytotoxic chemotherapy as both 1L (96.9%) and 2L (97.9%) palliative systemic treatment. The median PFS of 9.4 months observed with 1L systemic therapy (89.7% of whom received Gem and platinum) is contextualised by PFS values for 1L Cis/Gem of 8.0 months in the ABC-02 trial [6] and 5.7 months in the more recent TOPAZ-1 trial [8]. This suggests potentially increased chemo-responsiveness in this population versus an unselected population of patients with ABC, or that patients included in clinical trials have more regular imaging intervals and/or follow-up, and therefore progression may be detected somewhat sooner—the imaging interval in the TOPAZ trial was 6-weekly for the first 24 weeks and then 8-weekly thereafter, as opposed to 12-weekly in the ABC-02 trial and with standard-of-care treatment.

For 3L systemic treatment, 10.3% of patients received molecularly targeted therapies. This study’s long inclusion period (2006 to 2022) spans multiple therapeutic advances within the field of BTC—accordingly, the first included patient to receive a molecularly targeted agent did so in 2016, with all others having received them since 2018, reflecting the recent rise in their use. A statistically significant improvement in OS was seen for patients treated with molecularly targeted agents, despite the relatively small absolute number of patients who received one, suggesting a wide margin of superiority with their use. Agents targeting FGFR2 fusions and IDH1 inhibitors are currently approved for 2L use [11,12,13] and FGFR2 inhibitors are the subject of 1L clinical trials (infigratinib NCT03773302, pemigatinib NCT03656536); although only 3L use was observed in the present population, this direction of travel may enable a higher proportion of patients with the relevant aberrations to receive molecularly targeted therapy before disease- and chemotherapy-related toxicities render them unable to do so.

Other analyses have aimed to review outcomes for patients following three lines of palliative systemic therapy; Thol et al. determined a mOS1 of 18.2 months for 25 patients treated at a single German centre [26], versus 26.9 months for this population. A further analysis by Rizzo et al., presented in abstract form, found an mOS3 of 4.4 months and mPFS3 of 2.8 months (versus 6.4 and 3.1 months, respectively, for the present population) among 101 patients treated at three academic centres in Italy, with a similarly high proportion of included patients having the iCCA subtype [27]; this study specified chemotherapy as the 3L treatment, and hence, presumably did not include patients treated with targeted therapies, which may explain the differences in survival between studies.

Notably, some included patients received re-challenge Cis/Gem in line with institutional preference, including in 2L. The authors acknowledge that the definition of line of therapy in this instance is uncertain and debated. We elected to include such patients, as any other treatment commencing at the same point in time would certainly be considered 2L. Equally, it would seem remiss to discount any systemic therapy commenced for the purpose of controlling ABC, and which precludes other concurrent systemic therapy, from this analysis. For ovarian cancer, the well-established concept of a platinum-free interval may help guide the utility of re-challenge therapy; although an interval of 6 months in that setting is a typical cut-off after which re-challenge may be beneficial, this is still not universally agreed, and differences are expected between tumour types [28]. Platinum-free interval did not influence inclusion in the present study. Previous analyses, including those mentioned above, have not included data regarding the re-challenge of systemic therapy, reporting proportions of each treatment received in each line but not reporting any relationships between lines of treatment [24,26,29].

Despite significant recent advances, the majority of patients with ABC do not have a molecular aberration with a corresponding FDA-approved agent identified; hence, cytotoxic chemotherapy remains the mainstay of treatment for BTC as a whole, as it was for the present study population. Cytotoxic chemotherapy use in 3L was largely in line with currently approved 1L and 2L combinations, or their single-agent variants. A higher proportion of patients received single-agent cytotoxic chemotherapy in the 3L setting versus previous lines—29.9%, compared with 2% 1L and 9.3% 2L—which is consistent with an expected decline in performance status as patients’ disease progresses through lines of therapy [30]. Remarkably, however, 19.6% of patients in this study cohort did receive fourth-line palliative systemic therapy, and of these, nearly all received combination chemotherapy. This may reasonably be attributed to the more favourable biology of these patients’ disease. It may be relevant that chemotherapy dose reductions were not captured within this data collection, which is a limitation.

A majority of included patients exhibited the iCCA subtype, which is incongruent with the relative incidence rates between subtypes of BTC [2,3,4]; it is, however, consistent with the preponderance of FGFR2 fusions and IDH1 mutations within iCCA, which not only have approved targeted therapies, but are proposed as conferring favourable prognosis irrespective of receiving a targeted therapy, thus improving the likelihood of reaching 3L palliative systemic treatment [31]. Another reason for the preponderance of patients with iCCA may be that some such patients were referred to the included academic centres on a ‘second opinion’ basis, with a view to accessing molecular profiling, clinical trials and/or targeted therapy, potentially skewing the present population towards iCCA.

The current analysis did not suggest any differences in OS1 between anatomical subtypes of BTC (including iCCA versus non-iCCA) for included patients, despite post hoc data from 1L trials of Gem and Cis/Gem +/− the vascular endothelial growth factor inhibitor cediranib suggesting superior prognosis for the iCCA subtype even in the pre-molecularly targeted therapy era [32]. This may be due to the selected nature of the population—patients with extrahepatic disease are generally more prone to biliary obstruction with potential for consequent infection, treatment delays, and hence, potentially poorer prognosis. Patients with extrahepatic disease who survived to receive three lines of palliative systemic therapy are therefore unlikely to have encountered these complications, and hence prognosis may be more comparable between anatomical subgroups in the present population. A prior large retrospective analysis of clinical trials provides evidence for significantly poorer prognosis for GBC versus other BTC subtypes [33], which may account for the under-representation of GBC in this analysis. Possibly due to the above factors, there was highly differential representation of the anatomical subtypes in this population, with low absolute numbers in some cases; this limited analysis of the prognostic significance of anatomical subtype, where no clear signal was detected.

The authors note that due to the retrospective nature of data collection and unavailability of RECIST 1.1 measurements, response rate data were not collected for the present analysis. There was also variation in the proportion of patients who received 3L systemic therapy between participating centres, which is likely to reflect differences in practice more than variations in the accessibility of 3L systemic therapy; there may also be population differences in factors such as comorbid status, ECOG PS, and socioeconomic status to account for some of these differences. In this study, PFS was used to approximate time to treatment escalation. Treatment was given on progression and was not actively foregone in favour of surveillance in any patients; therefore, PFS should approximate this well, although the authors note that this remains an approximation.

Outcomes for patients with ABC receiving 1L and 2L palliative systemic therapy have been well-documented in other publications [6,7,24]; as such, the purpose of the current study was to define the less-described therapeutic options and outcomes for patients receiving 3L therapy in real-world practice. A wider review of a larger population of patients who received 1L and 2L treatment was felt to be unlikely to differ from previously available data.

There is clearly significant variation within this population in terms of baseline clinicopathological features, disease course, and treatments received. As such, the purpose of these data is not to provide a recommendation for a single preferred 3L regimen; such decisions should be informed by factors such as response to previous lines of treatment, treatment-free interval, ECOG PS, comorbid status, availability of clinical trials, and increasingly by molecular profiling to determine suitability for targeted therapies. For patients with no identified targetable alterations, randomized studies of a systemic therapy versus best supportive care may be a potential future trial design.

## 5. Conclusions

The wider availability of molecular profiling, combined with the development of more efficacious targeted therapies, is expected to produce more deviations from the established standard lines of chemotherapy treatment, as systemic therapy becomes increasingly personalised. Similarly, the proportion of patients with advanced BTC who receive 3L systemic therapy is likely to increase, in line with the higher absolute patient numbers seen later in the study period; factors driving this shift are likely to include a broader range of available targeted therapies, whose toxicity profile is typically non-overlapping with respect to cytotoxic chemotherapy, as well as the receipt of such targeted therapies earlier in a patient’s treatment course. A more complete understanding of tumour biology after the receipt of specific molecularly targeted therapies is likely to be necessary to optimise these novel treatment pathways. In conclusion, this relatively large multi-institutional retrospective study demonstrates that in select patients with ABC, systemic treatment is feasible and appears to be beneficial, and the presented efficacy values provide benchmarks for future trial design.

## Figures and Tables

**Table 1:** Patient characteristics.

	Number of Patients (% *)
Age at diagnosis—median (IQR)	61 (52–68)
Male gender	54 (55.6)
ECOG PS 0-1 **	95 (97.9)
Prior excisional surgery	39 (40.2)
Prior adjuvant chemotherapy	10 (10.3)
**Anatomical subgroup**	
Intrahepatic CCA	60 (61.9)
Perihilar CCA	13 (13.4)
Distal CCA	12 (12.4)
GBC	14 (14.4)
AVC	3 (3.1)
**First-line palliative systemic therapy received**	
Gem/platinum	87 (89.7)
5-FU/platinum	3 (3.1)
Other ***	7 (7.2)
**Second-line palliative systemic therapy received**	
Gem/platinum	31 (32.0)
5-FU/platinum	29 (29.9)
5-FU/irinotecan	18 (18.6)
Other ***	17 (17.5)
**Locoregional therapy received**	
SIRT	14 (14.4)
Palliative radiotherapy	10 (10.3)
Chemoembolisation	1 (1.0)

* Unless otherwise stated. ** At first oncology review. *** See Supplementary Table S3 for further information. IQR, inter-quartile range. ECOG PS, Eastern Co-operative Oncology Group Performance Status. CCA, cholangiocarcinoma. GBC, gallbladder carcinoma. AVC, ampulla of Vater carcinoma. Gem, gemcitabine. 5-FU, 5-fluorouracil. SIRT, selective internal radiation therapy.

**Table 2:** Third-line palliative systemic therapy received by patients with advanced biliary tract cancer.

	Number of Patients (%)
**Chemotherapy**	
5-FU/platinum	24 (24.7)
5-FU/irinotecan	13 (13.4)
Gem/platinum	12 (12.4)
Capecitabine or 5-FU	11 (11.3)
Paclitaxel or Docetaxel	10 (10.3)
Gem/capecitabine	4 (4.1)
Gem	4 (4.1)
Irinotecan	3 (3.1)
EOX	1 (1.0)
Raltitrexed	1 (1.0)
**Targeted or combination therapy**	
FGFR inhibitor	7 (7.2)
Ivosidenib	2 (2.1)
Sunitinib	2 (2.1)
APL 101	1 (1.0)
5-FU/platinum/cetuximab	1 (1.0)
Pembrolizumab/ramucirumab	1 (1.0)

5-FU, 5-fluorouracil. Gem, gemcitabine. EOX, epirubicin, oxaliplatin, and capecitabine. FGFR, fibroblast growth factor receptor.

## Data Availability

Not applicable.

## Supplementary Materials

The following supporting information can be downloaded at: https://www.mdpi.com/article/10.3390/cancers15113047/s1, Table S1: Year of receipt of first-line palliative systemic therapy; Table S2: Fourth-line palliative systemic therapy received by patients with advanced biliary tract cancer and progression through third-line palliative systemic therapy documented prior to death; Table S3: First- and second-line palliative systemic therapy received by patients with advanced biliary tract cancer defined as ‘other’ in Table 1.
