# Supplementary material for: Third-Line Palliative Systemic Therapy for Advanced Biliary Tract Cancer: Multicentre Review of Patterns of Care and Outcomes
Source: Cancers (Basel). 2023 Jun 3;15(11):3047. doi: 10.3390/cancers15113047 (PMC10252105; doi:10.3390/cancers15113047)
Supplement: Supplementary file 1 [file cancers-15-03047-s001.zip › cancers-2377461-supplementary.pdf]

Supplementary Materials:

Table S1. Year of receipt of first-line palliative systemic therapy.

Year 1st-Line Palliative Systemic Therapy Received	*Number of Patients*
2006	2
2007	3
2008	2
2009	4
2010	5
2011	5
2012	3
2013	10
2014	10
2015	8
2016	9
2017	6
2018	12
2019	7
2020	7
2021	1

Table S2. Fourth-line palliative systemic therapy received by patients with advanced biliary tract cancer and progression through third-line palliative systemic therapy documented prior to death.

	Number of Patients (n = 66)
5-FU/platinum	*5 (7.6%)*
5-FU/irinotecan	*6 (9.1%)*
Gem/platinum	*4 (6.1%)*
Gem	*3 (4.5%)*
RXC004 plus Nivolumab	*1 (1.5%)*

5-FU = 5-fluorouracil. Gem = gemcitabine.

Table S3. First- and second-line palliative systemic therapy received by patients with advanced biliary tract cancer defined as `other` in Table 1.

	Number of Patients
**First-line therapy**	
Gem	*2*
Cis/Gem plus durvalumab/placebo	*2*
Cis/Gem plus cediranib	*1*
EOX	*1*
5-FU/irinotecan	*1*
**Second-line therapy**	
Capecitabine	*6*
Gem/capecitabine	*5*
5-FU	*2*
Gem	*1*
Durvalumab plus tremelimumab	*1*
Regorafenib plus avelumab	*1*
Capecitabine/platinum	*1*

Gem = gemcitabine. 5-FU = 5-fluorouracil. EOX = epirubicin, oxaliplatin, capecitabine.
